# Impairment of chondrocyte biosynthetic activity by exposure to 3-tesla high-field magnetic resonance imaging is temporary

**DOI:** 10.1186/ar1991

**Published:** 2006-07-10

**Authors:** Ilse-Gerlinde Sunk, Siegfried Trattnig, Winfried B Graninger, Love Amoyo, Birgit Tuerk, Carl-Walter Steiner, Josef S Smolen, Klaus Bobacz

**Affiliations:** 1 Department of Internal Medicine III, Division of Rheumatology, Medical University of Vienna, Waehringer Guertel 18–20, A-1090 Vienna, Austria; 2 Department of Radiology, Medical University of Vienna, Waehringer Guertel 18–20, A-1090 Vienna, Austria; 3 Medizinische Universitätsklinik, Klinische Abteilung für Rheumatologie, LKH Graz, Auenbruggerplatz 15, 8036 Graz, Austria

## Abstract

The influence of magnetic resonance imaging (MRI) devices at high field strengths on living tissues is unknown. We investigated the effects of a 3-tesla electromagnetic field (EMF) on the biosynthetic activity of bovine articular cartilage. Bovine articular cartilage was obtained from juvenile and adult animals. Whole joints or cartilage explants were subjected to a pulsed 3-tesla EMF; controls were left unexposed. Synthesis of sulfated glycosaminoglycans (sGAGs) was measured by using [^35^S]sulfate incorporation; mRNA encoding the cartilage markers aggrecan and type II collagen, as well as IL-1β, were analyzed by RT–PCR. Furthermore, effects of the 3-tesla EMF were determined over the course of time directly after exposure (day 0) and at days 3 and 6. In addition, the influence of a 1.5-tesla EMF on cartilage sGAG synthesis was evaluated. Chondrocyte cell death was assessed by staining with Annexin V and TdT-mediated dUTP nick end labelling (TUNEL). Exposure to the EMF resulted in a significant decrease in cartilage macromolecule synthesis. Gene expression of both aggrecan and IL-1β, but not of collagen type II, was reduced in comparison with controls. Staining with Annexin V and TUNEL revealed no evidence of cell death. Interestingly, chondrocytes regained their biosynthetic activity within 3 days after exposure, as shown by proteoglycan synthesis rate and mRNA expression levels. Cartilage samples exposed to a 1.5-tesla EMF remained unaffected. Although MRI devices with a field strength of more than 1.5 T provide a better signal-to-noise ratio and thereby higher spatial resolution, their high field strength impairs the biosynthetic activity of articular chondrocytes *in vitro*. Although this decrease in biosynthetic activity seems to be transient, articular cartilage exposed to high-energy EMF may become vulnerable to damage.

## Introduction

The imaging of articular cartilage in clinical practice relies mainly on conventional radiography and ultrasound. In joint disorders with concomitant cartilage damage, imaging techniques that visualize the whole cartilage are desirable for diagnostic purposes. Magnetic resonance imaging (MRI) has been proposed to serve such an aim, because the MRI technique is unparalleled in its capacity to delineate the morphology and composition of articular cartilage [1].

In contrast to the planar images provided by conventional radiographic techniques, MRI permits the assessment of the whole articular surface of a joint. This allows one to evaluate cartilage defects and thinning in regions of the joint not visible to radiography or ultrasound, which also provides greater sensitivity to change. Currently, 1.5-tesla standard MRI devices are widely used; however, to improve the signal-to-noise ratio that would ultimately result in an increase in spatial resolution and contrast [2], higher field strengths of 3 T and more have been introduced and may replace 1.5-tesla machines in the near future.

However, MRI techniques require patients to be exposed to an intense electromagnetic field (EMF) of a strength not previously encountered. As radio frequency energy is absorbed more effectively at higher frequencies [3], safety concerns regarding possible interactions between high-energy EMFs of at least 3 T and living tissues were raised. Potential mechanisms could be a distortion in the orientation of macromolecules and membranes, effects on the conductibility of peripheral nerves, electrocardiographic or electroencephalographic alterations or effects on blood rheology; however, the vast majority of the scans have been performed without any evidence of sequelae to the patients and therefore the technique has been considered to be safe [4]. Nevertheless, there is a lack of studies on the direct influence of high-energy EMFs on living tissues including articular cartilage. In contrast, investigations of low-energy EMFs *in vitro *showed a direct stimulatory effect on cartilage matrix synthesis [5,6] and cell proliferation [7,8]. The clinical efficacy of these EMFs has also been shown in bone fracture healing [9,10]; moreover, low-energy EMFs have been used in the non-invasive therapy of degenerative joint diseases [11,12].

On the basis of these observations we wondered whether high-energy EMFs would have similar effects on the biosynthetic activity of articular cartilage. In the present study we investigated the influence of a 3-tesla EMF on the matrix biosynthesis of chondrocytes derived from bovine articular cartilage.

## Materials and methods

### Tissue culture and exposure to EMF

Hooves from 15 three-month-old calves and 8 adult steers were obtained from a local slaughterhouse (Steininger, Simondsfeld, Austria). Because the hooves and the metacarpophalangeal joints are not used in meat processing and constitute waste material, no ethics committee approval was required. Metacarpophalangeal joints were prepared by the removal of skin and appendages.

Experiments were performed by exposing either whole joints or cartilage explant cultures to the pulsed EMF. The samples were divided into two groups (control group and 'pulsed EMF' group). The specimens of the 'pulsed EMF' group were subjected either to a 3-tesla MRI device (Medical 3T MedSpec; Bruker, Ettingen, Germany) or a 1.5-tesla MRI device (MAGNETOM Vision/Plus; Siemens, Erlangen, Germany).

For whole-joint exposure, joints from juvenile animals were wrapped in plastic wrap. Thereafter the joints of the 'pulsed EMF' group were subjected to a pulsed EMF (constant 3-tesla and additional 0.0135-tesla pulsed field, pulse rate 0.5 s) for the duration of a standard knee-joint examination, namely 25 minutes. Controls were left unexposed. Directly after exposure to the EMF, the joints of both groups were opened aseptically; cartilage samples were obtained [13] and washed twice in PBS (GibcoBRL, Life Technologies, Paisley, Renfrewshire, UK). Afterwards, one part of these tissue explants was distributed into 24-well plates (Costar, Cambridge, MA, USA), in quadruplicate at 100 to 150 mg of cartilage wet weight per well, for isotope incorporation assays. The ratio of medium to tissue (1.5 ml per 100 mg of cartilage) was always kept constant [13,14]. The other part of the cartilage samples was used for mRNA isolation, alkaline phosphatase assays, and cell death assays; to this end, tissue was digested for 8 hours in 0.2% collagenase B (Roche Diagnostics GmbH, Penzberg, Germany) and filtered through a cell strainer (Falcon; Becton Dickinson Labware, Lincoln Park, NJ, USA) to remove debris and undissociated cell clusters. Evaluation of the chondrocyte number was performed after trypan blue staining in a Bürker–Türk chamber.

In some experiments, time-course analyses were performed after the exposure of cartilage explants to the 3-tesla MRI device or the 1.5-tesla device (using a standard protocol for routine knee-joint examination). Metacarpophalangeal joints of 3-month-old calves and adult steers were opened aseptically and cartilage samples (100 to 150 mg of cartilage wet weight) were grown in 24-well plates in quadruplicate in serum-free basal medium (BM). The serum-free BM consisted of DMEM/Ham's F-12 (1:1) with ITS plus culture supplement (Collaborative Biomedical Products, Bedford, MA, USA), α-ketoglutarate (100 μM), caeruloplasmin (0.25 U/ml), cholesterol (5 μg/ml), phosphatidylethanolamine (2 μg/ml), α-tocopherol acid succinate (0.9 μM), reduced glutathione (10 μg/ml), taurine (1.25 μg/ml), triiodothyronine (1.6 nM), hydrocortisone (1 nM), parathyroid hormone (0.5 nM), β-glycerophosphate (10 mM final concentration) and L-ascorbic acid 2-sulfate (50 μg/ml) (Sigma Chemical Co., St Louis, MO, USA) [15] and was shown to be appropriate for chondrocyte cultures by the method of Erlacher and colleagues [16]. The explant cultures were allowed to adjust to the culture settings for 24 hours. After the BM had been changed after 24 hours, half of the explant cultures were exposed to the EMFs exactly as described above; untreated cultures served as controls. Subsequently, the explants were subjected to [^35^S]sulfate incorporation assays, as well as RNA isolation directly after exposure to the EMF (day 0) as well as on days 3 and 6.

All cultures were maintained at 37°C in humidified air containing 5% CO_2_.

### Biosynthesis of macromolecules

Cartilage specimens were labelled in 1 ml of BM containing 20 μCi/ml of [^35^S]sulfate (carrier-free; Amersham, Little Chalfont, Buckinghamshire, UK) for 4 hours at 37°C. After radiolabeling, the explants were washed three times with ice-cold buffer (10 nM EDTA, 0.1 M sodium phosphate, pH 6.5) followed by digestion overnight in 1 ml of sodium phosphate wash buffer containing proteinase K (1 mg/ml) at 80°C. Unincorporated isotope was removed by Sephadex G-25 gel chromatography on a PD-10 column (Pharmacia Biotech, Piscataway, NJ, USA). Values were obtained by liquid-scintillation counting (1410 liquid-scintillation counter; Wallac Oy, Turku, Finland) of aliquots from void volume fractions and normalized to hydroxyproline content [17], because the basal turnover of the collagen network is known to be very low in articular cartilage [18,19] and was assumed to remain unaffected during the study period. Additionally, the DNA content of the digested samples was assessed with the use of bisbenzimide (Hoechst 33258; Sigma) in accordance with established protocols [20].

### Histologic analysis

Tissue punches including cartilage and subchondral bone from the control (*n *= 5) and 'pulsed EMF' (*n *= 5) groups were fixed in 10% formalin, embedded in paraffin and sectioned at 5 μm. After deparaffination the sections were stained with toluidine blue in accordance with standard protocols. To distinguish differences in metachromasia in the different sections, digitized images were analyzed for intensity of staining. By the use of Quantity One v. 4.5.2 software (Bio-Rad Laboratories, Hercules, CA, USA), we determined color densities of 20 randomly selected areas of both the pericellular and the territorial/interterritorial zones on a total cartilage area of 0.25 mm^2 ^for each specimen. The densities of the pericellular zones were then normalized to the densities of the territorial/interterritorial zones.

### RNA isolation and RT–PCR

For total RNA extraction, chondrocytes were isolated from cartilage explants of 100 to 150 mg wet weight per specimen in 0.2% collagenase B for 8 hours. The extraction of total RNA was performed with a commercially available kit (RNeasy; Qiagen, Valencia, CA, USA) in accordance with the manufacturer's protocols.

RT–PCR was used to determine the presence of aggrecan, type II collagen, osteocalcin (OC), osterix, runx2/cbfa1 and IL-1β mRNA. Total RNA (1 μg) from each sample was copied into cDNA in a 20 μl reaction by using the First-Strand cDNA Synthesis Kit (Amersham Biosciences). Aliquots of 1 μl were amplified in a 10 μl reaction mixture that contained 50 mM Tris-HCl pH 8.3, 2 mM MgCl_2_, 0.25% bovine serum albumin, 2.5% Ficoll 400, 5 mM tartrazine, 200 μM dNTPs, each primer at 1 μM, and 0.2 U *Taq *polymerase (Boehringer Mannheim, Mannheim, Germany). The reaction profile as employed here comprised an initial denaturation at 94°C for 2 minutes, followed by 35 cycles (aggrecan, IL-1β)/33 cycles (type II collagen)/32 cycles (osteocalcin, osterix, runx2/cbfa1)/25 cycles (β-actin) at 94°C for 45 seconds, 55°C for 45 seconds, and 72°C for 55 seconds, and an additional extension step of 5 minutes at 72°C after the last cycle. Amplification reactions were performed in an air thermal cycler (Mastercycler; Eppendorf AG, Hamburg, Germany). Reaction products were analyzed by electrophoresis in 1.5% agarose gels. The amplified DNA fragments were detected with a Fluorimager (Bio-Rad Laboratories) and band densities were calculated with Quantity One software (Bio-Rad Laboratories). Negative controls in which cDNA was omitted from the reaction, as well as positive controls (human articular cartilage for aggrecan and type II collagen, peripheral blood mononuclear cells for IL-1β, and bovine periosteum for osteocalcin, osterix and runx2/cbfa1) were run in parallel. Primer sequences used were as follows: aggrecan (470 bp), 5'-TCC CAG AAT CCA GCG GTG AGA G-3' (forward) and 5'-GCA CAG GGC TTG AGG ATT CG-3' (reverse) [21]; type II collagen (593 bp), 5'-TCG GGG CTC CCC AGT CGC TGG TG-3' (forward) and 5'-GAT GGA GAA CCT GGT ACC CCT GGA-3' (reverse) [22]; osteocalcin (362 bp), 5'-GAC AGA CAC ACC ATG AGA ACC-3' (forward) and 5'-CTA GCT CGT CAC AGT CAG GG-3' (reverse) [23]; osterix (358 bp), 5'-GCAGCTAGAAGGGAGTGGTG-3' (forward) and 5'-GCAGGCAGGTGAACTTCTTC-3' (reverse) [24]; runx2/cbfa1 (270 bp), 5'-CCCCACGACAACCGCACCAT-3' (forward) and 5'-CACTCCGGCCCACAAATC-3' (reverse) [25]; IL-1β (394 bp), 5'-AAA CAG ATG AAG AGC TGC ATC CAA-3' (forward) and 5'-CAA AGC TCA TGC AGA ACA CCA CTT-3' (reverse) [26]; β-actin (520 bp), 5'-TGT GAT GGT GGG AAT GGG TCA G-3' (forward) and 5'-TTT GAT GTC ACG CAC GAT TTC C-3' (reverse).

### Alkaline phosphatase activity

After digestion with collagenase, the isolated cells were distributed in 24-well plates in quadruplicate at a density of 10^5 ^cells/cm^2 ^and then sonicated in 500 μl of 0.1% Triton X-100 in distilled water. Aliquots (100 μl) of each sample were incubated with 100 μl of alkaline phosphatase substrate buffer (100 mM diethanolamine, 150 mM NaCl, 2 mM MgCl_2_) containing the soluble, chromogenic alkaline phosphatase substrate *p*-nitrophenyl phosphate (2.5 μg/ml) for 5 to 25 minutes at room temperature. The reaction was stopped with 50 μl of 1 M NaOH/0.1 M EDTA. Measurement was performed with an ELISA Reader (MR 7000; Dynatech, Guernsey, Channel Islands, UK) at 405 nm. Enzyme activity was expressed as nmoles of *p*-nitrophenol released, normalized to the protein content of the sample. Monolayer cultures of bovine periosteal cells incubated with 10% FBS (PAA Laboratories, Linz, Austria) served as positive controls. Total cellular protein was determined with the Bio-Rad protein assay in accordance with the manufacturer's instructions (Bio-Rad Laboratories GmbH, Munich, Germany); the absorbance of samples was measured at 550 nm.

### TdT-mediated dUTP nick end labeling and Annexin V assays

For cell death assessment we used the terminal deoxynucleotidyl-transferase (TdT)-mediated dUTP nick end labeling technique (TUNEL; *In Situ *Cell Death Detection Kit with fluorescein; Roche Diagnostics GmbH) and the Annexin V-FITS assay (Alexis Austria, Vienna, Austria). Cartilage samples were digested in collagenase B as described above. Chondrocytes (10^6 ^per sample) were cultured for a further 24 hours in 50 ml tubes in BM.

For TUNEL assays the cells were washed three times with PBS and finally suspended in 100 μl of PBS in Micronic Tubes (Micronic System, Lelystad, The Netherlands). Cells were fixed and permeabilized with the Fix&Perm cell permeabilization kit (An der Grub Inc., Kaumberg, Austria). In brief, after the addition of 100 μl of fixation solution and incubation for 1 hour at 20°C the samples were centrifuged at 300 *g *for 10 minutes. The supernatant was discarded and 100 μl of the permeabilization solution was added to the tubes. Subsequently, 50 μl of TUNEL reaction mixture was added and the suspension was incubated for 1 hour at 37°C. Thereafter, samples were analyzed on a FACScan (Becton Dickinson, Evembadegen, Belgium) by dual-color immunocytofluorimetry [27].

Additionally we investigated cell death in cartilage sections from the control (*n *= 5) and 'pulsed EMF' (*n *= 5) groups by using the In-Situ Cell Death Detection Kit with fluorescein (Roche Diagnostics GmbH) in accordance with the manufacturer's instructions. The sections were evaluated by fluorescence microscopy (Axioskop 2 mot plus; Zeiss, Oberkochen, Germany).

For the Annexin V-FITS assays, 10^6 ^chondrocytes per sample were washed three times with PBS and subsequently suspended in 195 μl of PBS. Annexin V-FITS labeling buffer (5 μl) was added and the samples were left to rest for 10 minutes before resuspension in 200 μl of binding buffer. A 10 μl aliquot of propidium iodide solution was added. The samples were analyzed by dual-color cytofluorimetry [27].

### Statistical analysis

Data are expressed as means ± SD. Statistical analysis was performed with Student's *t *test to compare treated with untreated samples. Statistical significance was defined as *p *< 0.05.

## Results

### The exposure of whole joints to a 3-tesla EMF affects cartilage biosynthetic activity

Because low-energy EMFs have been reported to increase cartilage matrix synthesis, we investigated the effects of a high-energy 3-tesla EMF on the biosynthetic activity of articular cartilage. Metacarpophalangeal joints of 3-month-old calves were subjected to a 3-tesla EMF (3 T constant plus a 0.0135 T pulse every 0.5 s) has been corrected. As a functional reflection of biosynthesis, cartilage explants were subjected to [^35^S]sulfate incorporation assays to evaluate the neosynthesis of sulfated glycosaminoglycans (sGAGs). Moreover, their RNA was isolated and subjected to RT–PCR to determine changes in gene expression for the major cartilage proteins aggrecan and collagen II as well as for the cytokine IL-1β.

**Figure 1:**
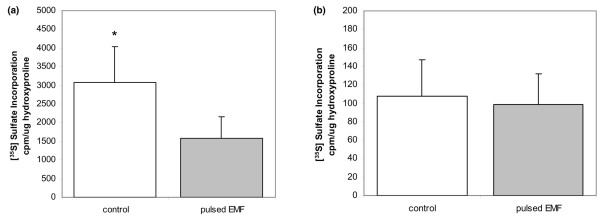
Effects of a 3-tesla electromagnetic field on glycosaminoglycan synthesis. **(a) **Bovine metarcarpophalangeal joints (*n *= 5) were exposed to a 3-tesla electromagnetic field (EMF); untreated joints (*n *= 5) served as controls (control). Cartilage samples were obtained aseptically from the joints and labeled with [^35^S]sulfate for 4 hours. The incorporated radiolabel into the newly synthesized matrix macromolecules was then measured and normalized to hydroxyproline content of the explants. Results are means and SD. *p < 0.0002 versus 'pulsed EMF'. **(b) **Subsequently, sulfated glycosaminoglycans in the supernatant of the explant cultures were measured. Results are means and SD.

**Figure 2:**
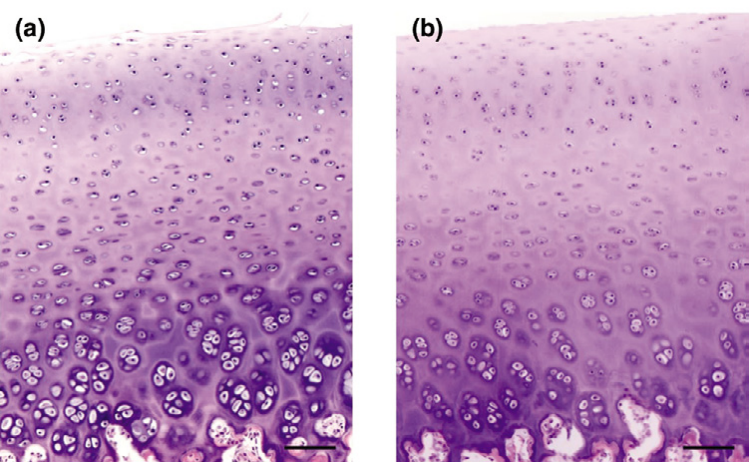
Histological sections of bovine articular cartilage and histochemical comparison. Metacarpophalangeal joints derived from 3-month-old calves were subjected to a 3-tesla electromagnetic field (EMF) (*n *= 5) or were left untreated (*n *= 5). Thereafter, tissue punches including cartilage and subchondral bone were prepared for histological analysis and stained with toluidine blue in accordance with standard protocols. The figure shows two representative sections: **(a) **control; **(b) **cartilage after exposure to a 3-tesla EMF. Scale bars, 100 μm.

Exposure to the 3-tesla EMF resulted in an unexpected decrease in total sGAG synthesis compared with unexposed controls. In the control group a mean [^35^S]sulfate incorporation rate of 3,068.6 ± 973.1 (mean ± SD) c.p.m./μg of hydroxyproline was measured, whereas in the 'pulsed EMF' sample group we observed a marked decrease in isotope uptake (1,588.9 ± 559.1 c.p.m./μg of hydroxyproline). This decrease was highly significant compared with the control group (*p *< 0.0002) and reflected a decrease in sulfate incorporation of 48% (Figure 1a). In addition, a histochemical comparison of cartilage sections after staining with toluidine blue revealed a less intense staining of the pericellular zones in the 'pulsed EMF' group than in controls (Figure 2). When we normalized the densities of the pericellular zones to those of the territorial/interterritorial zones we found a significant decrease (*p *< 0.0002) in the 'pulsed EMF' group (1.36 ± 0.21 integrated optical density) in comparison with the control group (1.47 ± 0.18 integrated optical density).

**Figure 3:**
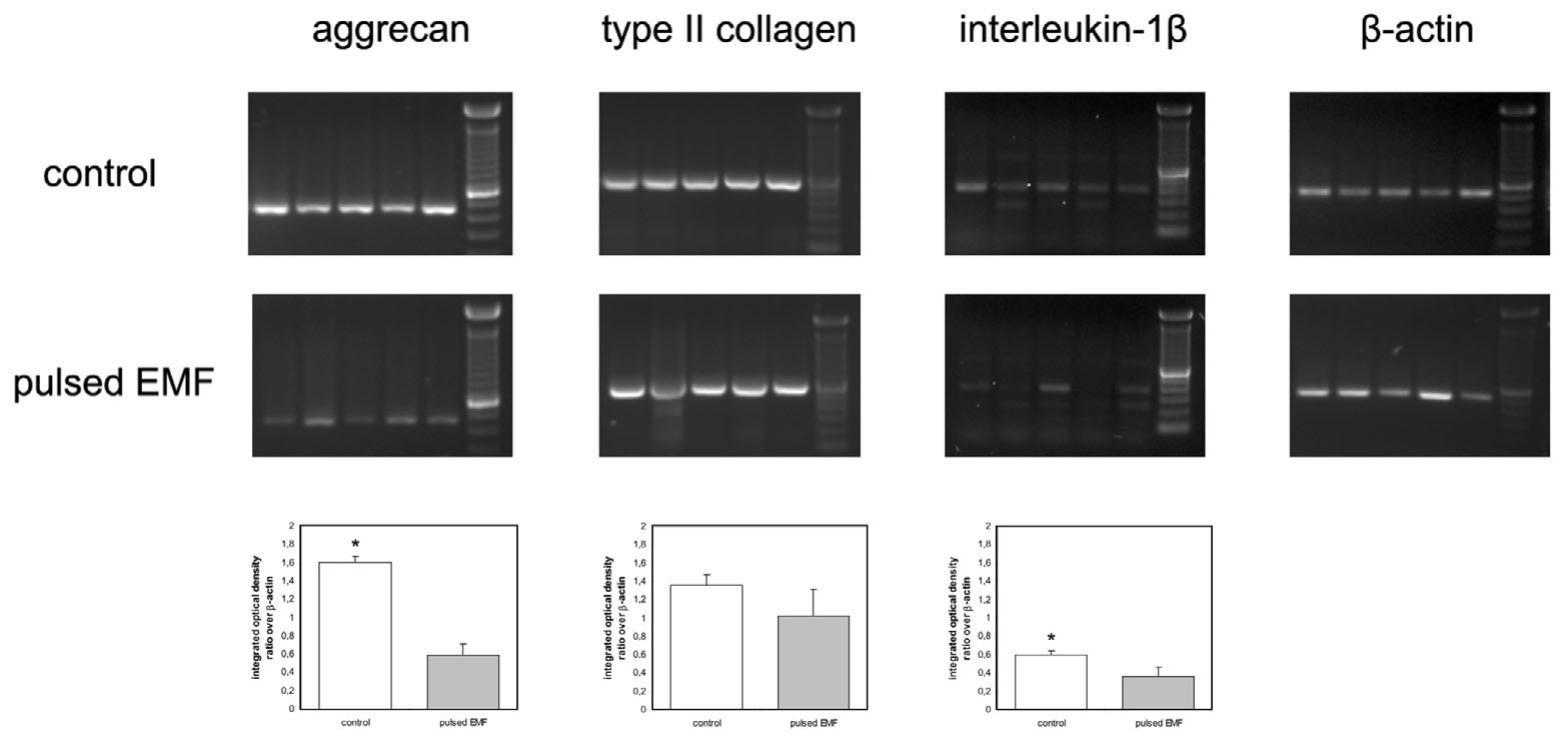
Expression of the cartilage markers aggrecan and collagen type II and also of IL-1β. Unexposed cultures served as negative controls (control). mRNA was obtained as described in the Materials and methods section. The presence of aggrecan, type II collagen and IL-1β, was detected by RT–PCR. The bar graphs show the integrated optical density of the bands after normalization to β-actin. Values are means and SD. Aggrecan: *p < 0.0002 control versus 'pulsed EMF'; IL-1β: **p < 0.02 control versus 'pulsed EMF'.

Given the detrimental effects of the 3-tesla EMF on sGAG synthesis in articular cartilage, RT–PCR was performed to quantify the gene expression of both aggrecan and type II collagen, the major cartilage matrix components. As shown in Figure 3, in the 'pulsed EMF' group aggrecan was highly downregulated compared with untreated controls (*p *< 0.0002), supporting the data on [^35^S]sulfate incorporation. The mRNA expression of type II collagen was not significantly changed after exposure to the 3-tesla EMF (*p *= 0.09; Figure 3).

To determine whether these findings reflected a decrease in cellular activity or an increase in catabolic activity, the expression of IL-1β, an important cytokine in cartilage biology [28] was measured, as was the release of sGAGs into the culture supernatant. The exposure to the EMF did not lead to an overexpression of IL-1β mRNA; rather – and in accordance with the decrease in overall biosynthetic activity – we found a decrease in IL-1β expression in the 'pulsed EMF' group (*p *< 0.02) when band densities were normalized to those of β-actin (Figure 3). In line with this finding, there was no significant difference in sGAG content in the culture supernatant (*p *= 0.27) between the 'pulsed EMF' group (196.7 ± 15.6 c.p.m./μg of hydroxyproline) and the control group (215.8 ± 18 c.p.m./μg of hydroxyproline) and thereby no evidence for a major loss of sGAGs from the cartilage matrix (Figure 2b).

These results suggest a decrease in biosynthetic activity, according to the sGAG synthesis rate, of articular chondrocytes after exposure to the 3-tesla EMF rather than a loss of matrix macromolecules driven by catabolic events induced by IL-1β or other molecules.

### Exposure to a 3-tesla EMF does not induce cell death in articular chondrocytes

A decrease in cartilage biosynthesis could be caused by cell death of resident cells, leading to a decrease in chondrocyte numbers, which could have explained the results described above. To determine a possible effect of a 3-tesla EMF on cell survival, we assessed cell death rate and cellular DNA content in chondrocytes after exposure to a 3-tesla EMF.

**Figure 4:**
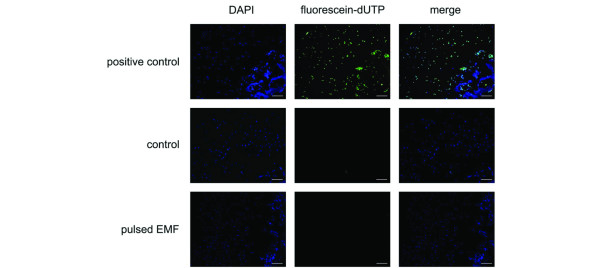
Detection of cell death in articular chondrocytes after being subjected to a 3-tesla electromagnetic field. Sections from articular cartilage (*n *= 5) were assessed for cell death rate after exposure to a 3-tesla pulsed electromagnetic field (EMF) by using a direct TUNEL labeling assay and were compared with a control group (*n *= 5) that was left unexposed. Fluorescein-dUTP (green) and nuclear staining with 4',6-diamidino-2-phenylindole (DAPI; blue) reflect no difference in cell death rate between the control and the 'pulsed EMF' group. The figure shows one representative experiment. DNAse-1-treated articular cartilage served as positive control. Scale bars, 100 μm.

Chondrocytes subjected to the EMF showed no increased cell death rate compared with controls, as shown by cytofluorimetry (Annexin V; control group 7.8 ± 0.9 versus 'pulsed EMF' group 8.8 ± 2.8% gated cells; TUNEL, control group 0.8 ± 0.4 versus 'pulsed EMF' group 0.9 ± 0.2% gated cells). In addition, in histological sections we found no increased cell death rate in the 'pulsed EMF' group compared with the control group by labeling of DNA strand breaks with the use of TUNEL technology (Figure 4). Furthermore, there was no difference in the DNA content of the cartilage samples (*p *= 0.9) between the control group (0.9 ± 0.27 ng/ml per μg of hydroxyproline) and the 'pulsed EMF' group (0.9 ± 0.37 ng/ml per μg of hydroxyproline).

### Impairment of chondrocyte activity induced by the 3-tesla EMF is transient

**Figure 5:**
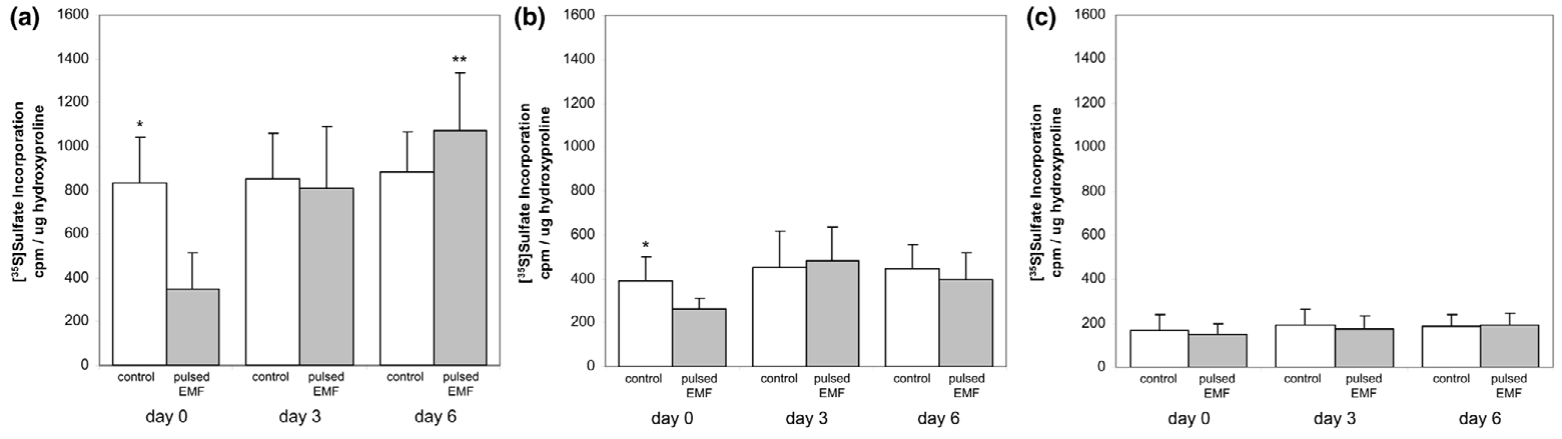
Time course of effects of a 3-tesla electromagnetic field on glycosaminoglycan synthesis. Cartilage explants derived from bovine metacarpophalangeal joints were incubated in serum-free basal medium (BM) and subjected to an electromagnetic field (EMF). The control group was left unexposed. The total proteoglycan synthesis rate was evaluated on days 0, 3 and 6 after exposure. Values were normalized to hydroxyproline content of the explants. Values are means and SD. **(a) **Exposure of juvenile bovine cartilage (*n *= 5) to a 3-tesla EMF. The white columns represent the unexposed controls (control), the grey columns the samples exposed to the EMF (pulsed EMF). *p < 0.0001 control versus 'pulsed EMF', **p < 0.02 'pulsed EMF' versus control. **(b) **Exposure of adult bovine cartilage (*n *= 3) to a 3-tesla EMF. The white columns represent the unexposed controls (control), the grey columns the samples exposed to the EMF (pulsed EMF). *p < 0.008 control versus 'pulsed EMF'. **(c) **To evaluate the effects of an EMF of less than 3 T, adult bovine cartilage (*n *= 5) was exposed to a 1.5-tesla EMF. The white columns represent the unexposed controls (control), the grey columns the samples exposed to the EMF. No significant difference between the groups could be determined.

According to the cell death and DNA data, there was no detectable cell damage after exposure to EMF. We therefore investigated whether the pulsed EMF led to a persistent decrease in the metabolic activity of cartilage or whether the decreased metabolic rate recovered from the effects of the pulsed EMF, regaining its basal biosynthetic activity. For this purpose we used cartilage explant cultures from metacarpophalangeal joints of calves (young group) and adult steers (old group) which were subjected to the pulsed EMF; controls were left unexposed. On days 0, 3 and 6 after exposure the rate of newly synthesized matrix macromolecules was measured. In line with the above data obtained after the exposure of whole joints to the pulsed EMF, we found a marked decrease in total sGAG synthesis in both groups on day 0 after exposure to the EMF: in the young group, control samples yielded a mean isotope uptake rate of 836 ± 205.8 c.p.m./μg of hydroxyproline, whereas in the EMF-treated samples the rate was 352 ± 160.9 c.p.m./μg of hydroxyproline (48% decrease, *p *< 0.0001; Figure 5a). Cartilage samples derived from adult steer joints also displayed a significant decrease in sGAG biosynthesis after exposure to the pulsed EMF (control group, 391.1 ± 107.8 c.p.m./μg of hydroxyproline; 'pulsed EMF' group, 262.2 ± 50.9 c.p.m./μg of hydroxyproline; *p *< 0.008; Figure 5b), indicating a 33% decrease in isotope uptake and thus shows the susceptibility to EMF also of adult cartilage.

Importantly, however, articular chondrocytes recovered from the EMF effects: on day 3, 'pulsed EMF' juvenile cartilage synthesized a mean of 810.9 ± 281.9 c.p.m./μg of hydroxyproline (control group 851.9 ± 205.6 c.p.m./μg of hydroxyproline, *p *= 0.65), and on day 6 the respective values were 1,070.8 ± 266.4 and 880.1 ± 187.9 c.p.m./μg of hydroxyproline (*p *< 0.02; Figure 5a). This ultimate increase in the 'pulsed EMF' group might be interpreted as a reactive enhancement of sGAG production after initial biosynthetic regress. Unlike the metabolically highly active juvenile cartilage, adult tissue showed no such 'rebound phenomenon': while the decrease in biosynthetic activity of the samples paralleled the overall results of young cartilage on day 0, biosynthesis was comparable to controls on day 3 (control group, 454.4 ± 161.5 c.p.m./μg of hydroxyproline; 'pulsed EMF' group, 481.5 ± 151.1 c.p.m./μg of hydroxyproline; *p *= 0.81) and on day 6 (control group, 446.5 ± 111 c.p.m./μg of hydroxyproline; 'pulsed EMF' group, 394.5 ± 123.3 c.p.m./μg of hydroxyproline; *p *= 0.45; Figure 5b).

To determine whether these effects were a unique property of high-energy fields, such as a 3-tesla EMF, we tested the influence of a 1.5-tesla EMF on matrix macromolecule neosynthesis in adult bovine cartilage. Interestingly, in contrast to the 3-tesla EMF, the 1.5-tesla field did not influence cartilage metabolic activity (Figure 5c).

**Figure 6:**
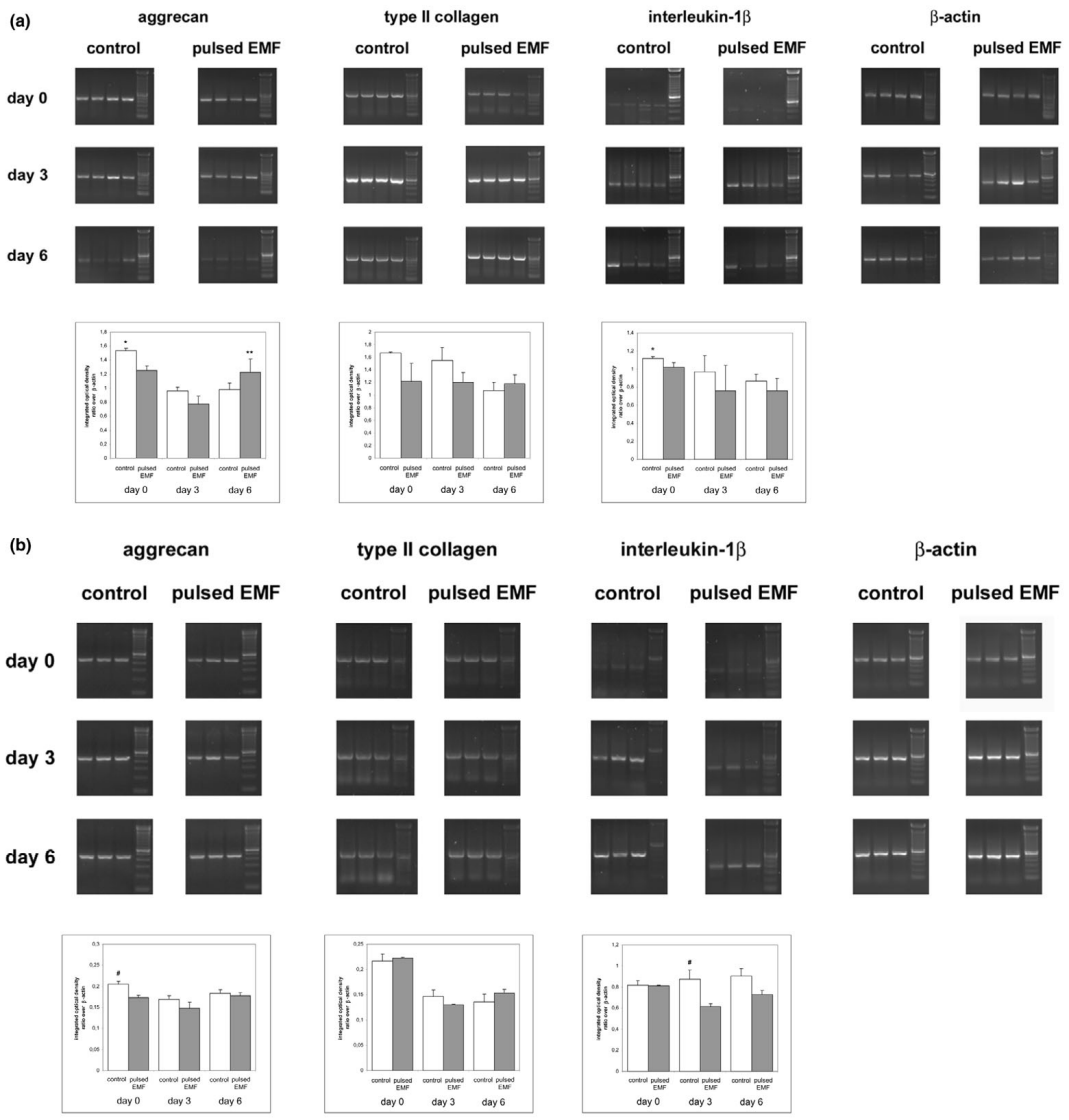
Time course of expression of aggrecan, type II collagen and IL-1β. The endogenous expression of aggrecan, type II collagen and IL-1β was assessed with RT–PCR on days 0, 3 and 6 after exposure to a 3-tesla electromagnetic field (EMF). Unexposed cultures served as controls (control). The integrated optical density of the bands was determined and normalized to that of the β-actin bands as shown in the bar graphs. Values are means and SD. **(a) **mRNA was obtained from juvenile bovine cartilage samples (*n *= 4) Aggrecan: *p < 0.009 control versus 'pulsed EMF'; **p < 0.05 'pulsed EMF' versus control; IL-1β: *p < 0.03 control versus 'pulsed EMF'. **(b) **mRNA was obtained from adult bovine cartilage samples (*n *= 3). Aggrecan: ^#^*p *< 0.02 control versus 'pulsed EMF'; IL-1β: ^#^*p *< 0.02 control versus 'pulsed EMF'.

In parallel with sGAG synthesis, the mRNA expression of aggrecan was assessed after exposure of explant cultures to the 3-tesla EMF. In line with the data obtained for whole-joint EMF exposure, in both the young (Figure 6a) and adult (Figure 6b) groups aggrecan gene expression was significantly downregulated on day 0 after being subjected to the 3-tesla EMF (*p *< 0.009 and *p *< 0.02, respectively). The subsequent normalization and even increase in sGAG synthesis at the end of the culture period observed in the young group was also reflected by a significant increase in aggrecan gene expression on day 6 (*p *< 0.05).

Whereas the endogenous expression of IL-1β was decreased on day 0 in the young group, the old group displayed a delayed response because IL-1β levels decreased on day 3 of the culture period. Collagen type II mRNA did not change significantly in the young or in the old group (Figure 6).

Chondrocyte DNA content remained unaffected during the experimental period in the young and in the old group (data not shown).

### Osteogenesis is not induced after exposure to the 3-tesla EMF

Differentiation of fetal chondrocytes toward an osteogenic phenotype under the influence of a high-energy EMF has been described previously [29]. To determine the effects of a 3-tesla EMF on possible osteogenic differentiation, we determined the endogenous expression of early and late markers of osteogenic differentiation. We investigated the expression of osterix, runx2/cbfa1 and osteocalcin and found no increase in mRNA expression levels for osterix (control, 0.77 ± 0.02 integrated optical density/β-actin; 'pulsed EMF', 0.69 ± 0.09 integrated optical density/β-actin), runx2/cbfa1 (control, 0.79 ± 0.07 integrated optical density/β-actin; 'pulsed EMF', 0.82 ± 0.06 integrated optical density/β-actin) or osteocalcin (control, 0.98 ± 0.08 integrated optical density/β-actin; 'pulsed EMF', 0.99 ± 0.2 integrated optical density/β-actin). Additionally, alkaline phosphatase activity was measured showing no increase in enyzmatic activity (control, 0.0069 ± 0.0017 nmol of *p*-nitrophenyl phosphate per minute per microgram of protein; 'pulsed EMF', 0.0073 ± 0.0016 nmol of *p*-nitrophenyl phosphate per minute per microgram of protein; *p *= 0.6) under the influence of the 3-tesla EMF (not shown).

## Discussion

The present study revealed the unexpected result of a significant decrease in matrix macromolecule synthesis of cartilage after exposure to a 3-tesla EMF. These data are based on the sGAG synthesis rate in cartilage as well as gene expression profiling of articular chondrocytes, demonstrating a decrease in aggrecan mRNA synthesis, whether exposed to the high-energy EMF as whole joint or as a cartilage explant. Because lower-energy EMFs have not been shown to induce a decrease in cartilage biosynthetic activity in this and previous studies [5,6,30], the results obtained seem to be a consequence of the exposure to a 3-tesla high-energy EMF.

It has been hypothesized that an EMF might act like a mechanical load that causes a movement of fluid, which contains charged particles, relative to the solid matrix structures such as proteoglycans and collagens with their fixed charges [9,10,31,32]. This fluid flow generates an electrical potential, the so-called 'streaming potential' [33,34], which transduces mechanical stress into an electrical phenomenon capable of stimulating chondrocytes to synthesize matrix components. Physiologically, mechanical stresses on cartilage range from about 0 to 20 MPa [35] and stimulate the synthesis of matrix constituents [36]; exceeding this threshold causes physical damage to the cartilage [37-39]. Taking these facts into account, a low-energy EMF may mimic mechanical stresses within physiological amplitudes, potentially leading to cellular stimulation [5-8], whereas a high-energy EMF is likely to resemble stresses above physiological ranges, thereby initiating an inadequate flow of electrolytes and charges that ultimately impair cartilage activity. This assumption is fostered by our observations of a marked decrease in anabolic activity, as shown by sGAG synthesis and aggrecan gene expression. The studies of Lee and colleagues [40] and Trinidade and colleagues [41], who showed an impaired cartilage activity after applying mechanical stresses, are in line with our findings.

It is noteworthy that collagen type II expression was not appreciably changed, which may be attributed to the very low basal turnover of the collagen network [18,19]. Beyond that, the release of sGAGs to the supernatant remained unchanged from that in the controls, indicating no major catabolic activity. Though not catabolic by itself, our inability to find increased expression of IL-1β upon exposure of cartilage to high-energy EMF is in line with the above results. A limiting factor to our study could be the fact that tissue manipulation and digestion can affect chondrocyte gene expression. Although the results from the RT–PCR analysis support the [^35^S]sulfate incorporation data, the effects of the exposure to the EMF may have been masked by changes in gene expression resulting from enzymatic digestion and associated events. However, the decrease in both anabolic and catabolic activity led us to speculate that a high-energy EMF may to some extent compromise the biosynthetic activity and/or function of articular chondrocytes.

Although it is known that mechanical stress contributes to the induction of chondrocyte cell death [42-44], in our experimental settings we found no difference in cell death rates between EMF-exposed and control samples, either in TUNEL or in Annexin V assays, which excludes cell death and a consequent decrease in chondrocyte numbers as a cause of the findings. Additionally, the 3-tesla EMF had no impact on the DNA content of the cartilage specimens, making a loss of chondrocytes very unlikely as a reason for the impaired biosynthetic activity.

Whether the results obtained also relate to the situation *in vivo *and in humans will have to be confirmed in similar analyses of human cartilage or in animal studies. However, it is also unknown whether this impairment of chondrocyte activity has any implication for the development of cartilage damage as seen in osteoarthritis. To address these questions, animal studies or studies in humans, for instance by the delayed gadolinium-enhanced MRI of cartilage (dGEMRIC) technique, will be necessary.

The ability of articular chondrocytes to recover from mechanical strains has been proposed previously [39,45]. When investigating the effects of the 3-tesla EMF over a period of 6 days, we did in fact find a recovery of cartilage biosynthetic activity. Furthermore, we tested whether there was a difference in the susceptibility to a high-energy EMF between young and old cartilage. The results on sGAG and aggrecan mRNA synthesis obtained on day 0 resembled the data from our initial measurements, in both young and old samples. Subsequently, the chondrocytes regained their biosynthetic activity over the course of time. At the end of the culture period an increase in sGAG/aggrecan mRNA production was found in the young group but not in the adult group after EMF exposure. This observation may be seen as a 'rebound phenomenon' caused by a higher metabolic rate of these cells in young cartilage compared to adult cartilage. In line with a lower metabolic activity of old chondrocytes [46-48], such a rebound was not seen in tissues from aged cartilage. Our data therefore suggest that the effects of a 3-tesla EMF are transient and articular chondrocytes recover from the initial impairment.

## Conclusion

A high-energy EMF potentially impairs the biosynthetic activity of articular chondrocytes; this effect is temporary, as shown under the *in vitro *conditions employed here. Because no such influence on articular cartilage could be seen after exposure to a 1.5-tesla EMF, this impact on cellular activity seems to be a characteristic of 3-tesla high-energy EMFs.

Our data therefore indicate that the assessment of musculoskeletal structures with 3-tesla MRI devices may be accompanied by a transient disturbance in chondrocyte function after exposure to the EMF. Given that cartilage with reduced biosynthetic activity may be deficient in its repair capacity, patients may have to be advised to minimize their physical activities for up to 72 hours after high-field MRI examination to prevent possible damage to the articular cartilage.

## Abbreviations

BM = basal medium; bp = base pairs; EMF = electromagnetic field; IL = interleukin; MRI = magnetic resonance imaging; PBS = phosphate-buffered saline; RT–PCR = reverse transcriptase-mediated polymerase chain reaction; sGAG = sulfated glucosaminoglycan; TUNEL = TdT-mediated dUTP nick end labelling.

## Competing interests

The authors declare that they have no competing interests.

## Authors' contributions

IGS contributed to the study conception and design, drafted the manuscript, and performed cell culture experiments, sulfate incorporation assays, alkaline phosphatase assays and RT–PCR analysis. ST provided the 3-tesla MRI device and the appropriate device settings. WBG contributed to the study conception and design. LA conducted cell culture experiments, sulfate incorporation assays, alkaline phosphatase assays and RT–PCR analysis. BT performed histological sectioning and histochemical staining as well as TUNEL staining. CWS conducted Annexin V and TUNEL assays. JSS reviewed the manuscript critically and gave final approval of the version to be published. KB set up the study conception and design and the preparation of the manuscript. All authors read and approved the final manuscript.
